# Liver Injury with Features Mimicking Autoimmune Hepatitis following the Use of Black Cohosh

**DOI:** 10.1155/2009/918156

**Published:** 2010-01-10

**Authors:** Grace Guzman, Eric R. Kallwitz, Christina Wojewoda, Rohini Chennuri, Jamie Berkes, Thomas J. Layden, Scott J. Cotler

**Affiliations:** ^1^Department of Pathology, University of Illinois, Chicago, IL 60612, USA; ^2^Department of Medicine, University of Illinois, Chicago, IL 60612, USA; ^3^Department of Pathology, Case Western Reserve University, Cleveland, OH 44106, USA

## Abstract

There are a growing number of cases detailing acute hepatic necrosis in patients taking black cohosh (*Cimicifuga racemosa*), an over-the-counter herbal supplement for management of menopausal symptoms. Our aim is to illustrate two cases of liver injury following the use of black cohosh characterized by histopathological features mimicking autoimmune hepatitis. Both patients reported black cohosh use for at least six months and had no evidence of another cause of liver disease. Their liver biopsies showed a component of centrilobular necrosis consistent with severe drug-induced liver injury. In addition, the biopsies showed characteristics of autoimmune-like liver injury with an interface hepatitis dominated by plasma cells. Although serum markers for autoimmune hepatitis were not particularly elevated, both patients responded to corticosteroids, supporting an immune-mediated component to the liver injury. Liver injury following the use of black cohosh should be included in the list of differential diagnoses for chronic hepatitis with features mimicking autoimmune hepatitis.

## 1. Introduction

Black cohosh (*Cimicifuga racemosa*) is an herbal supplement that is sold over-the-counter for management of menopausal symptoms, menstrual irregularities, and arthritis. There are now a number of manuscripts indicating liver injury from black cohosh [1–7]. Here, we present two patients who developed a drug-induced liver injury following the use of black cohosh characterized by centrilobular necrosis and histologic features mimicking autoimmune hepatitis (AIH) who improved with corticosteroid therapy.

## 2. Case Studies

The case series was approved by the Institutional Review Board at the University of Illinois at Chicago.

**Case 1**

A 42-year-old woman presented to her primary care provider with a two-month history of progressive malaise, nausea, vomiting, and dizziness. On physical examination, she had mild icterus and no stigmata of chronic liver disease. Her only comorbidity was hypothyroidism. Her medications included black cohosh, which she was taking for 6 months, and levothyroxine 0.05 mg daily. She discontinued black cohosh when her symptoms began. She had no family history of liver or autoimmune disease. She denied any alcohol or drug use. Her total bilirubin level was 3.1 mg/dL (normal 0.8–1.2), alanine transaminase (ALT) 1457 U/L (normal 5–40), aspartate transaminase (AST) 696 U/L (normal 10–40), alkaline phosphatase (ALP) 94 U/L (normal 30–120 U/L), and international normalized ratio (INR) 1.2 (normal 0.8–1.2). Further laboratories showed no serological evidence of active hepatitis A, B, or C infection. Serological titers for acute cytomegalovirus (CMV) and Epstein Barr virus (EBV) were negative. Antinuclear antibody (ANA) was 1 : 40 and smooth muscle antibody was modestly elevated at 1 : 80. Serum iron was 169 *μ*g/dL (normal: 50 to 170 *μ*g/dL for women), ferritin was 642 ng/dL (normal: 15–200 ng/mL for females), and hemochromatosis gene analysis was negative. Ceruloplasmin was in the normal range.

**Figure 1:**
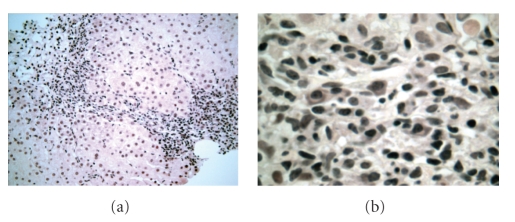
This liver biopsy from Case 1 displays chronic hepatitis remarkable for its dense interface activity with cholangiolar proliferation (1a) (×200) and sheets of plasma cells and apoptosis (1b) (×600).

Four months after her initial presentation, the liver enzymes remained elevated with an ALT of 334 U/L, AST 229 U/L, and a total bilirubin of 1.4 mg/dL. A liver biopsy was performed which prompted a referral to the liver center at the University of Illinois. The liver biopsy was remarkable for necrosis around terminal hepatic venules, lobular disarray, mild ballooning, apoptosis, Kupffer cell reactivity, mild cholangiolar proliferation, and interface hepatitis remarkable for numerous plasma cells and eosinophils. A repeat antismooth muscle antibody was normal at 1 : 20. Prednisone treatment was initiated. A follow-up liver biopsy was performed after three months. At this time, the liver histology demonstrated chronic hepatitis with fibroinflammatory expansion of portal tracts by moderate plasma cell rich interface hepatitis, rare periportal hepatocyte apoptosis, and mild cholangiolar proliferation (Figures 1(a) and 1(b)). The patient was started on azathioprine. Her liver enzymes returned to normal values within five months with an ALT of 30 U/L, AST of 29 U/L, ALP 36 U/L, and a total bilirubin of 0.9 mg/dL. A repeat liver biopsy after fifteen months showed marked improvement with only minimal portal lymphocytes and no other significant histopathological changes. The liver function test values remained normal at 15 months and at 40 months following the acute episode. The clinicopathological features of Case 1 are illustrated in Tables 1, 2, and 3.

**Case 2**

A 53-years-old woman presented to our liver center for evaluation of elevated liver enzymes detected at a health screening fair four months earlier. She had no risk factors for viral hepatitis, no family history of liver disease, and denied any alcohol consumption. Although she was initially asymptomatic, she developed right upper quadrant abdominal pain, fatigue, and lower extremity edema by the time of her presentation. Her medical history was remarkable only for irritable bowel syndrome. Her medications within the past year included dicyclomine for abdominal cramps as needed, an herbal supplement which contains soy protein and black cohosh for menopausal symptoms, and occasional over the counter nonsteroidal anti-inflammatory drugs (NSAIDs) for pain. She discontinued these medications on the advice of her primary care physician. Laboratories showed a total bilirubin of 2.0 mg/dL, ALT 443 U/L, AST 478 U/L, ALP 188 U/L, and an INR of 2.0. Other notable laboratory values included negative serological evidence of active hepatitis A, B, and C viral infection, undetectable hepatitis C virus RNA by PCR, iron 137 *μ*g/dL, ferritin 1527 ng/mL, negative hemochromatosis gene analysis, and nonsignificant ceruloplasmin level. Serological tests for antinuclear, antismooth muscle, antiliver kidney microsomal, and antimitochondrial antibodies were negative. An ultrasound demonstrated patent portal and hepatic veins and a normal appearing liver. Her liver biopsy was remarkable for centrilobular necrosis with moderate cholangiolar proliferation and plasma cell rich interface and lobular hepatitis.

The patient was started on 40 mg of prednisone daily. Liver function test values improved to a total bilirubin of 1.4 mg/dL, ALT of 60 U/L, AST 50 U/L, and ALP 153 U/L after two weeks and normalized after five weeks of therapy. The clinicopathological features of Case 2 are illustrated in Tables 1, 2, and 4.

## 3. Application of the Naranjo Causality Score for Adverse Drug Reactions

The Naranjo causality score for adverse drug reactions [8] was applied to the cases described here. As shown in Table 5, the current case studies individually achieved a Naranjo score of 3 and were both categorized as “possible adverse drug reaction”, in a scale encompassing a minimum score of zero, or “doubtful adverse drug reaction” to a maximum score of ≥9, or “definite adverse drug reaction.”

## 4. Application of the Hennes-Simplified Criteria for Diagnosis of Autoimmune Hepatitis (AIH)

While the current case studies showed histopathological features of autoimmune hepatitis, neither fulfilled a diagnosis of “probable AIH” or “definite AIH” based on the Hennes simplified criteria for diagnosis of AIH [10]. Briefly, Case 1 had an ANA titer of up to 1 : 80 (score 2), no available immunoglobulin titers (no score), a liver histology compatible with AIH (score 1), and absence of viral hepatitis (score 2), with a total score of 5. Case 2 had a score of 1-0-1-2 = 4, on a scale where a score of ≥6 was designated “probable AIH”, and ≥7 as “definite AIH.”

## 5. Discussion

Botanical dietary supplements are used commonly in the United States and are generally perceived to be safe [11]. In one study, nearly one half of persons reported use of at least one agent to their primary provider [12]. A major difficulty in studying black cohosh is uncertainty regarding the composition of the various black cohosh products. Black cohosh itself contains a number of secondary compounds including phenolic derivatives and triterpene glycosides [13]. Phytoestrogens were postulated to play a role in black cohosh binding to estrogen receptors, but the presence of these compounds is controversial. In one study, chromatographic analysis of 11 commercial black cohosh products showed that three were actually Asian *Actea* species [13]. An analysis of the remaining eight products found variability in the composition of triterpene glycosides and phenolic constituents. More importantly, not a single product contained a phytoestrogen. Thus, the mechanism by which black cohosh might impact on alleviating menopausal symptoms is not well understood. Other possibilities include binding to yet-to-be-defined receptors or effects on the central nervous system [14]. Whether black cohosh is effective in controlling vasomotor symptoms of menopause is controversial as well. The American College of Obstetrics and Gynecology included black cohosh as a possible remedy for the vasomotor symptoms of menopause [15]. A large randomized-blinded trial of one-year duration found no difference between black cohosh and placebo in controlling vasomotor symptoms [16]. Moreover, a recent review did not recommend long-term use due to the absence of safety evaluation [17]. 

There are a growing number of case reports of hepatotoxicity in patients taking black cohosh (Table 6) [1–7]. Most described acute hepatic necrosis, although three articles of them detailed a case with clinical features of autoimmune hepatitis after black cohosh use [1, 2, 5]. The clinicopathological features of the cases in the current series have a fascinating combination of findings. Both patients presented with an acute hepatitis displaying prominent centrilobular necrosis consistent with severe drug-induced liver injury. While autoantibodies were low titer or absent, the histology showed characteristics of autoimmune hepatitis with a plasma cell rich interface and lobular hepatitis. In addition, both patients responded to corticosteroids, supporting an immune mediated component to the liver injury.

Determining the risk of black cohosh hepatotoxicity is further complicated by variability in both dosage and number of other botanical herbal supplements contained in some preparations. As an example, in the case reported by Lontos [4], the preparation of black cohosh included 4 other botanicals: ground ivy, golden seal, gingko, and oat seeds. While liver injury remains unreported with the use of the last 3 botanicals, ground ivy contains a known hepatotoxin, pulegone. This case culminated in liver failure despite discontinuation of the herbal supplement [4]. 

Drug interactions might further potentiate negative outcomes resulting from hepatoxicity due to herbal supplements. That subjects in 3 of 5 reported cases of autoimmune-like liver injury following consumption of black cohosh (Table 6) also had concomitant intake of synthetic thyroid hormone, levothyroxine, merits attention [2, 5]. Herbal supplements such as venencapsan and black cohosh, and prescription drugs specifically rosiglitazone, ritonavir, and valproic acid, have had reported drug interactions with levothyroxine [1, 9, 18–22].

A 2008 review by Mahady assigned all known case reports of black cohosh-induced liver injury under the category of “possible causality” [9]. As earlier stated, both cases of the current study were also assigned “possible causality” by Naranjo scale [8]. Although no clinical or animal pharmacokinetic or toxicological information were identified in the Mahady review, it led to the inclusion of a cautionary statement on black cohosh products which was not previously required [9]. Despite such development, the use of botanical dietary supplements is generally viewed by the public as safe [11]. Providers are encouraged to screen patients about their consumption of botanical herbal supplements and to assess for any evidence of liver injury [1]. Timely discontinuation of hepatotoxic agents and, in some cases, early initiation of immunotherapy might prevent significant liver injury or could even be life saving [4]. 

The validity of reported cases of liver injury due to black cohosh illustrated in Table 6 has been disputed. Concerns that led to this uncertainty included cases in which the timing of liver injury occurred at a relatively short interval following exposure to black cohosh [2, 6, 7], presence of comorbidities such as cholelithhiasis [6] or concomittant use of drugs other than black cohosh that could have potentially triggered the adverse drug reaction [3, 4, 7], or the notion that some of the reported cases [2], including Case 1 of the current study (also reported by Mahady [23]), were indeed examples of autoimmune hepatitis rather than adverse drug reactions. Nonetheless, application of the Naranjo causality score to these case reports found possible cause for adverse drug reaction [8]. Further, autoimmune hepatitis is one of the known outcomes of drug-induced liver injury. The immune mechanism is precipitated by a drug or a metabolite acting as a hapten covalently binding to host cellular protein, converting into an immunogen [24] and thereby eliciting an autoimmune response. Outcomes illustrating an association of chronic hepatitis with autoimmune features developing following the use of the botanical supplement black cohosh need to be emphasized.

## 6. Conclusion

Here we described two cases of liver injury following the use of black cohosh characterized by chronic hepatitis with centrilobular necrosis and an interface activity dominated by plasma cells. While the histopathology in both cases was compatible with autoimmune hepatitis, clinical evidence for an autoimmune etiology was lacking. Nonetheless both cases improved upon withdrawal of the drug and immunosuppressive therapy, indicating that there was at least in part a drug-induced immunological basis to the liver injury. 

The current case studies, and three other published cases of liver injury following the use of black cohosh, share similar histological characteristics [1, 2, 5]. Hepatic manifestations following the use of black cohosh should therefore be added to the list of differential diagnoses of hepatic necrosis culminating in chronic hepatitis mimicking autoimmune hepatitis.

## Figures and Tables

**Table 1:** Current case studies: clinical features and medications.

Clinical features	Case 1	Case 2
Age (years)	42	53
Gender	Female	Female
Signs	Mild icterus	Increased LFTs
Symptoms	Progressive malaise, nausea	RUQ** abdominal pain, fatigue
	Vomiting, dizziness	Lower extremity edema
Stigmata of CLD	Absent	Absent

Medications	Black cohosh	OTC with soy and black cohosh

Indication	Menopausal symptoms	Menopausal symptoms
Dose	Not specified	Not specified
Duration	6 months	8 months

Other medications	Levothyroxine	Dicyclomine; NSAID

Indication	Hyperthyroidism	GI cramps; pain
Dose	0.05 mg daily	Not specified
Duration	Chronic	As needed

LFTs: Liver function tests.

RUQ: Right upper quadrant.

CLD: Chronic liver disease.

OTC: Over the counter drug.

NSAID: Nonsteroidal anti-inflammatory drug.

**Table 2:** Current case studies: past medical history, serological tests, and other pertinent laboratory data.

		Case 1	Case 2
Past medical history	Viral or non-viral hepatitis	Absent	Absent
	Alcohol consumption	Absent	Absent

Serological tests	Anti-HAV IgM	Absent	Absent
	Anti-HAV total	Absent	Absent
	HBV surface antigen	Absent	Absent
	Anti-HBV core	Absent	Absent
	Anti-HBV surface	Absent	Absent
	Anti-HCV	Absent	Absent
	EBV Ca IgM	Absent	Absent
	EBV Ca IgG	Absent	Absent
	EBV Na IgG	Absent	Absent
	EBV IgG	Absent	Absent
	Anti-CMV IgM	Absent	Absent

HCV RNA PCR		Negative	Negative

Other laboratory values	Iron (normal: 50–70 *μ*g/dL for women)	169 *μ*g/dL	137 *μ*g/dL
	Ferritin (normal: 15–200 ng/mL in women)	642 ng/mL	1527 ng/mL
	HFE gene analysis	Negative	Negative
	Ceruloplasmin	Normal	Normal

**Table 3:** Duration of Illness and black cohosh use, liver function tests, autoimmune markers, histology, and treatment.

Case 1						
*Time of use to illness:* 6 mo**↓						

*Time of use to discontinuation:* 6 mo**↓						

*Time of presentation to the liver *clinic*		*↓				

*Duration of illness in months*	2	4	7	12	15	40
*Treatment*	—	Prednisone	Azathioprine	—	—	—

*Liver function tests (normal)*						
Total bilirubin (0.8–1.2 mg/dL)	3.1	1.4	1.8	0.9	1.0	0.8
ALT (5–40 U/L)	1457	334	321	30	39	37
AST (10–40 U/L)	696	229	236	29	37	33
ALP (30–120 U/L)	94	68	66	36	38	37
*INR * (0.8–1.2)	1.2	1.17	—	0.95	—	0.92

*Autoimmune antibodies*						
Antinuclear antibody	1 : 40	1 : 20	—	—	—	—
Antismooth muscle antibody	1 : 80	—	—	—	—	—
*Liver biopsy findings *	—	Acute hepatitis	Chronic hepatitis	—	Biopsy at 15 mo.	

*Lobular*				
Necrosis	—	Central & portal	—	—	Absent	
Infiltrates	—	Mixed with eosinophils	Plasmacytic	—	Absent	
Other findings	—	Disarray, ballooning Apoptosis		—	No significant abnormality	

*Portal and periportal*				
Cholangiolar proliferation	—	Moderate	Moderate	—	Absent	
Infiltrate severity	—	Moderate	Moderate	—	Minimal	
Infiltrate predominant type	—	Mixed with eosinophils	Plasmacytic	—	Lymphocytes	
*Fibrosis *	—	Fibronecrosis	Fibronecrosis	—	Absent	

**Table 4:** Duration of Illness and black cohosh use, liver function tests, autoimmune markers and histology.

Case 2			
Time of use to illness: 8 m o n t h s*	*↓		

Time of use to discontinuation: 8 m o n t h s*	*↓		

Time of presentation to liver c l i n i c*		*↓	

*Duration in months*	4	4.5	5

*Treatment*		Prednisone	

*Liver function tests (normal values)*			
Total bilirubin (0.8–1.2 mg/dL)	2.0	1.4	0.6
ALT (5–40 U/L)	443	60	30
AST (10–40 U/L)	478	50	38
ALP (30–120 U/L)	188	153	98
*INR * (0.8–1.2)	2.0	—	1.3

*Autoimmune antibodies*			
Anti-nuclear antibody	Negative	1 : 20	—
Anti-smooth muscle antibody	Negative	—	—
*Liver biopsy findings *		—	—

*Lobular*			
Necrosis	Central	—	—
Infiltrates	Plasmacytic	—	—
Other findings	Disarray, apoptosis	—	—

*Portal and periportal*			
Cholangiolar proliferation	Moderate	—	—
Infiltrate severity	Moderate	—	—
Infiltrate predominant type	Plasmacytic	—	—
*Fibrosis *	Fibronecrosis	—	—

**Table 5:** Naranjo [8] Causality Scale for Adverse Drug Reactions.

Question/Scoring-Yes/No/Do not know or unavailable		Case 1	Case 2
1	Are there previous conclusive reports on this reaction? 1/0/0	0	0
2	Did the adverse event appear after the suspected drug was given? 2/−1/0	2	2
3	Did the adverse reaction improve when the drug was discontinued or a specific antagonist was given? 1/0/0	1	1
4	Did the adverse reaction appear when the drug was readministered? 2/−1/0	0	0
5	Are there alternative causes that could have caused the reaction? −1/2/0	−1	−1
6	Did the reaction reappear when a placebo was given? −1/1/0	0	0
7	Was the drug detected in any body fluid in toxic concentrations? 1/0/0	0	0
8	Was the reaction more severe when the dose was increased/ increasing, or less severe when the dose was decreased? 1/0/0	1	1
9	Did the patient have a similar reaction to the same or similar drugs in any previous exposure? 1/0/0	0	0

	Total score	3	3

Scoring: >9 : definite adverse drug reaction (ADR).

5–8 : probable ADR.

1–4 : possible ADR.

0 : doubtful ADR.

**Table 6:** Similarities and differences among current case studies and published cases of black cohosh hepatotoxicity.

Case reports	Whiting Case 1*	Whiting Case 2*	Lontos*	Levitsky*	Cohen*	Lynch*	Chow	Nisbet	Current study Case 1^##^	Current study Case 2
Age/gender	47F	43F	52F	50F	57F	54F	51F	50F	42F	53F

Botanical dietary contents	Black cohosh	Black cohosh, skullcap#, Valerean, Passionflower	Black cohosh, ground ivy#, golden seal, Gingko, oat seed	Black cohosh	Black cohosh	Black cohosh	Black cohosh	Black cohosh	Black cohosh	Black cohosh, soy

Other drugs					Levothyroxine	Levothyroxine			Levothyroxine	Dicyclomine

Serologies indicating presence of other liver diseases				Absent		Absent	Absent		Absent	Absent

Lab data indicating presence of other liver diseases				Absent		Absent	Absent		Absent	Absent

Presenting symptom	Jaundice		ALF	ALF		ALF	Jaundice	Acute hepatitis	Acute hepatitis	RUQ pain

Main liver biopsy finding				Acute hepatitis		Acute hepatitis	Massive liver necrosis		Centrilobular necrosis	Centrilobular necrosis

Lobular infiltrate						Mixed + plasma cells	Mononuclear infiltrate		Plasma cytic infiltrate	Lymphop-lasmacytic infiltrate
Other lobular findings				Necrosis		Bridging necrosis	Preserved bile ducts, collapsed parenchyma		Lobular disarray	Lobular disarray

Therapy				Prednisone	Immuno-therapy	Prednisone			Prednisone & azathioprine	Prednisone

Intervention	OLT			OLT		OLT	OLT	Withdrawal of black cohosh	Withdrawal of black cohosh	Withdrawal of black cohosh

Outcome		Recovery	LF s/p d/c black cohosh	Recovery	Recovery	LF death hemorrhage	Recovery	Recovery	Recovery	Recovery
AIH like histology					Present	Present	Present		Present	Present

Serological marker for AIH				Absent	Absent	Absent	Absent		Absent	Absent

Issues disputing validity of black cohosh hepatoxicity	Drug use for only 6 days	Multi-drug product	Multi-drug product	Multi drug use Ethanol, NSAID & cyclovir	7 day use; maybe AIH			2 weeks exposure; cholelithiasis	Maybe AIH	

*Cases adapted from Table 1 Lynch [5]; ALF: Acute liver failure; RUQ: Right upper quadrant pain.

OLT: Orthotopic liver transplant; LF: Liver failure.

^#^Herbal remedies known as hepatotoxic; ^##^Also reported by Mahady [9]; AIH: Autoimmune hepatitis.
